# Study on the Rheological Optimization and Multiscale Verification of the Asphalt Rejuvenator

**DOI:** 10.3390/ma18132925

**Published:** 2025-06-20

**Authors:** Shanglin Song, Zhen Wang, Xiupeng Yao, Meng Guo, Haihong Zhang, Hongbin Chen, Fukui Zhang

**Affiliations:** 1 Gansu Provincial Transportation Research Institute Group Co., Ltd., Lanzhou 730070, China; 18193106299@163.com (S.S.); 13893606919@163.com (H.Z.); 17801052381@163.com (H.C.); 2 Key Laboratory of Civil Engineering Safety and Durability of China Education Ministry, Department of Civil Engineering, Tsinghua University, Beijing 100084, China; 3 Scientific Observation and Research Base of Transport Industry of Long Term Performance of Highway Infrastructure in Northwest Cold and Arid Regions, Dunhuang 736200, China; 4 Beijing Municipal Road and Bridge Building Materials Group Co., Ltd., Beijing 100176, China; wangzhen_seu2006@163.com; 5 State Key Laboratory of Bridge Engineering Safety and Resilience, Beijing University of Technology, Beijing 100124, China; yaoxp@emails.bjut.edu.cn; 6 The Key Laboratory of Urban Security and Disaster Engineering of Ministry of Education, Beijing University of Technology, Beijing 100124, China; 7 Gansu Provincial Highway Development Group Co., Ltd., Lanzhou 730070, China; 13993109118@163.com

**Keywords:** RAP (recycled asphalt pavement), rejuvenator, oil-like materials of aromatic hydrocarbons, road performance

## Abstract

The use of Reclaimed Asphalt Pavement (RAP) is a sustainable strategy to conserve natural resources, reduce environmental pollution, and lower construction costs. However, aged asphalt in RAP suffers from oxidation and loss of light fractions, increasing stiffness and brittleness. A key scientific challenge is how to effectively restore the performance of aged asphalt while maintaining cost efficiency. In this study, a novel asphalt rejuvenator was developed to address this issue. The rejuvenator consists of 6% aromatic oil-like materials to replenish light components, 1.52% plasticizer to enhance ductility, and 0.3% modifier A to improve adhesion, with a total dosage of 7.82% by the mass of the aged binder. The rejuvenator meets the requirements of Chinese specifications. The performance evaluation was conducted at both asphalt binder and mixture scales. The results show that the rejuvenator significantly improves low-temperature cracking resistance and medium-temperature fatigue performance of aged binders, although it slightly reduces high-temperature rutting resistance. When applied to asphalt mixtures with 45% RAP, the rejuvenated mixtures exhibited enhanced low-temperature performance. A comparative analysis with commercial rejuvenators confirmed the developed product’s competitive performance and economic benefit. This study provides technical insight into rejuvenator design and addresses critical challenges in RAP recycling for sustainable pavement engineering.

## 1. Introduction

At present, economic and environmental sustainability has become a hot issue in the transport industry [1]. Asphalt pavement will experience aging under the combined action of environmental loads and vehicle loads [2,3] and lead to cracking and other pavement diseases, which in turn affects the road surface aesthetics and driving safety [4]. At this point, pavement maintenance will generate large quantities of recycled asphalt pavement (RAP) [5]. Rejuvenated asphalt pavement is a widely recognized technology. RAP can replace a certain percentage of the asphalt binder and aggregate required for paving, leading to resource recovery and reduced production costs [6,7,8]. However, the technology has some drawbacks [9]. RAP contains a large amount of aged asphalt binder, which reduces the bonding properties of the asphalt mixture. It can lead to premature damage to asphalt pavements [10]. Aging significantly increases the complex modulus of the asphalt binder and enhances its sensitivity to temperature and frequency changes [11]. There are five main types of asphalt pavement regeneration techniques: plant-mixed thermal regeneration, in situ geothermal regeneration, plant-mixed cold regeneration, in situ cold regeneration, and full-depth thermal regeneration [12]. Rejuvenators can reduce the hardness of rejuvenated asphalt mixtures. It is an additive used to restore aged asphalt binders in RAP [13].

The process of aging and the regeneration of asphalt binders has been well studied. Transferring asphalt binder components from asphaltene to colloid is the root cause of asphalt binder deterioration [14,15]. The regeneration mechanism of RAP involves the addition of lightweight components to the aged asphalt binder, with the objective of replenishing the volatiles and harmonizing the chemical compositions in order to restore the binder’s performance [16,17,18]. Aging asphalt binders can be rejuvenated with materials containing high light-oil components [19,20,21,22]. Yan et al. [23] showed that the chemical compositions of the asphalt binder and the rejuvenator are the two key factors affecting the effectiveness of the regeneration of aged asphalt binders. Determining the optimum rejuvenator dosage can effectively restore the performance of aged asphalt binders [24]. Yan [25] showed that an ordinary rejuvenator can only replenish the lightweight components in the asphalt binder and cannot replenish the failed SBS (Styrene-Butadiene-Styrene) modifier, and the performance of the rejuvenated asphalt binder is not up to the level of an unaged asphalt binder. Ding [26] showed that the SBS-modified asphalt binder has a better aging resistance, and the appropriate dosage of rejuvenator can make the regeneration of the aging asphalt binder performance meet the specification requirements. The regeneration mechanisms of different sources of biological regenerators vary considerably due to their different compositions. Plant-derived rejuvenators restore aged asphalt binders primarily through diffusion and fusion. Animal-source and most algae-source rejuvenators are rich in nitrogen; the molecular forces between the molecular layers of the aged asphalt binder and then depolymerize the aggregated macromolecules to restore the aged asphalt binder [27,28]. Most of the bio-oils have a positive effect on improving the colloidal stability of asphalt binders and they will affect the rheological properties of asphalt binders. The interaction of bio-oils with an asphalt binder hinders asphaltene aggregation and leads to the depolymerization of asphaltene aggregates [29]. The existing rejuvenator is mainly through the supplementation of an asphalt binder in the light component to achieve the effect of regeneration; the regeneration effect needs to be further improved. Low-temperature cracking resistance is an important test criterion for rejuvenated asphalt mixtures with a high RAP dosage. Chen et al. [30] found that a low RAP dosage (30%) did not affect the low-temperature performance of rejuvenated asphalt mixtures. However, at a high RAP dosage, the low-temperature properties of asphalt mixtures were significantly reduced. AI-Qadi et al. [31] also pointed out that the RAP dosage (>25%) increases the stiffness of asphalt mixtures and makes the mixture more susceptible to cracking, leading to failure. As a result, many states and agencies do not allow RAP dosages higher than 40 percent in hot rejuvenated asphalt mixtures [32,33]. In this paper, the RAP dosage is 45%, and the purpose is to promote the application of rejuvenated asphalt mixtures with a high dosage of RAP and verify the regeneration effect of the developed asphalt rejuvenator.

There are many types of asphalt rejuvenators, and the common reclamation agents mainly include different types of vegetable oils and waste engine oils [34,35,36]. Most of them have unstable properties and the raw materials are more difficult to obtain. Commercial asphalt rejuvenators are generally expensive. To reduce the cost of road construction and improve the regeneration effect of RAP, this paper develops an inexpensive liquid asphalt high-efficiency rejuvenator. Oil-like materials of aromatic hydrocarbons are one of the basic raw materials for petrochemical products. They are rich in lightweight components, which can soften the aged asphalt binder. Moreover, oil-like materials of aromatic hydrocarbons are inexpensive. In this research, the base oil of the rejuvenator was preferred to be oil-like materials of aromatic hydrocarbons by testing. Furthermore, the addition of modifier A will serve to enhance the adhesion between the asphalt binder and aggregate while simultaneously improving the low-temperature crack resistance of rejuvenated asphalt mixtures. Finally, the road performance of the developed rejuvenator was verified from both the asphalt binder and mixture scales.

## 2. Objectives of the Study

The main objective of this paper is to develop a new type of liquid rejuvenator and evaluate its recovery effect on the performance of aged asphalt binders and asphalt mixtures through formula confirmation while conducting economic analysis to verify its practical application value. Through this study, we hope to effectively activate aged asphalt binders, improve the utilization rate of recycled asphalt pavement (RAP) materials, reduce the consumption of new materials, and promote resource recycling and sustainable development. In addition, the economic analysis of the system has clarified the cost-effective advantages of the rejuvenator, providing a scientific basis for its large-scale promotion and application.

## 3. Materials and Methods

### 3.1. Introduction to the Preparation and Testing of Rejuvenated Asphalt Binders

#### 3.1.1. Test Raw Materials

There are two types of asphalt binder: the Pen.70 virgin asphalt binder (VAB, Cangzhou, China) and SBS-modified asphalt binders, and the fundamental indexes are shown in Table 1. In addition, the Pen.70 VAB is only used for mixture scale tests. The reason for the introduction of the Pen.70 VAB in the road performance verification is that both the Pen.70 VAB and SBS-modified asphalt binders (Beijing, China) are used in existing asphalt pavements. The testing method is based on the China Code specification [37]; the standardized values of each indicator refer to the China Code specification [38] (JTG F40-2004).

#### 3.1.2. Preparation Method of Rejuvenated Asphalt Binder

The preparation method of the rejuvenated asphalt binder is as follows:

(1) Unaged asphalt binder sample preparation: A certain mass of SBS-modified asphalt binder is heated to the flow state in a high-temperature oven at 170 °C. The asphalt binder is then poured into the mold.

(2) Preparation of aged asphalt binder: First, the asphalt binder is aged for 5 h (the mass of the sample is 50 g, and the aging temperature is 163 °C) using the thin film oven test (TFOT) for short-term aging, and then the standard aged asphalt binder samples are prepared by aging the PAV for 20 h (aging temperature is 100 °C, and pressure is 2.1 MPa) for long-term aging.

(3) Preparation of rejuvenated asphalt binder: Firstly, 500 g of the aged SBS-modified asphalt binder is heated to the flow state in the oven at 170 °C and then moved to the constant temperature oil bath at 170 °C and slowly added to a certain amount of rejuvenator. The sample is stirred with a high-torque electric stirrer at a speed of 600 r/min for 60 min, and finally, the sample of the rejuvenated asphalt binder is prepared by standing for 5 min.

#### 3.1.3. Introduction to Test Methods for Asphalt Binder Scale

(1) Complex modulus master curve test

A dynamic shear rheometer (DSR) is used to conduct frequency scanning tests to evaluate the dynamic viscoelastic properties of original, aged, and rejuvenated asphalt binders at different temperatures and frequencies. This frequency scanning test selects an aluminum parallel plate with a diameter of 25 mm; the spacing between the upper and lower plates is set to 1 mm. The angular frequency range is set to 100~0.1 rad/s (logarithmic growth mode), the temperature is set to 35 °C, 45 °C, and 55 °C, and the strain control mode is 1%. According to the time–temperature equivalence principle, the complex modulus curves at various temperatures can be translated horizontally, and the complex modulus master curves based on the reference temperature (45 °C in this test) can be obtained, which can represent the rheological characteristics of the asphalt binder at a wider frequency. Choosing 45 °C as the reference temperature is to understand better and predict the performance of asphalt materials in practical applications while ensuring the consistency and comparability of experimental data.

(2) Multiple Stress Creep Recovery (MSCR) test

The Multiple Stress Creep Recovery (MSCR) test is also performed using the DSR. A 25 mm diameter aluminum parallel plate is selected, the spacing between the upper and lower plates is set to 1 mm, and the test temperature is 64 °C. The asphalt binder was loaded under 0.1 kPa stress condition for 1 s, after which no stress was applied for 9 s, and the process was repeated 10 times. The method was then carried out under 3.2 kPa stress conditions for a total test time of 200 s. During road service, asphalt mixture will withstand a wide range of stresses from extremely light to heavy. A total of 0.1 kPa represents a relatively low stress level and can be used to simulate situations with low traffic flow or low temperatures; 3.2 kPa corresponds to a higher stress level, which is closer to the actual situation under high-temperature conditions or high-traffic loads. To quantitatively evaluate the high-temperature performance of different rejuvenated asphalt binders, there are two crucial viscoelastic parameters: the average unrecoverable creep flexibility Jnr (the unit is kPa^−1^) and the average creep recovery rate R, which are calculated according to Equations (1) and (2), respectively:

(1) $$J_{nr}=\frac{\left(\varepsilon_{u}-\varepsilon_{0}\right)}{10\sigma }$$

(2) $$R=\frac{\left(\varepsilon_{p}-\varepsilon_{u}\right)}{10\left(\varepsilon_{p}-\varepsilon_{0}\right)}\times 100\%$$

where $\varepsilon_{0}$ is the initial strain during the creep per cycle (%); $\varepsilon_{p}$ is the peak strain during the creep per cycle (%); $\varepsilon_{u}$ is the residual strain after the recovery of each cycle (%); *σ* is the stress level applied in the test (kPa).

(3) Glover–Rowe (G–R) parameter test

The G–R parameter proposed by Rowe well evaluates the fatigue cracking resistance of asphalt binders at medium temperatures [39]. The acquisition of this index also requires a DSR test. An aluminum parallel plate with a diameter of 8 mm is selected and the complex shear modulus master curve is fitted using the Sigmoidal model to improve the accuracy [40]. The complex shear modulus corresponds to 0.005 rad/s in the master curve and the phase angle is substituted into Equation (3) for a calculation to obtain the G–R parameter. It has been established that the G–R parameter when reaching 180 kPa or above will result in the production of fatigue cracking in the asphalt binder. Furthermore, research has indicated that when the parameter reaches 450 kPa, severe fatigue cracking will occur [41].

(3) $$G\text{-}R=\frac{\left|G^{*}\right|\cdot {\left(\cos\delta \right)}^{2}}{\sin\delta }$$

where G* is the complex shear modulus (kPa); δ—phase angle (rad).

(4) Low-temperature performance test (4 mm plate test)

The low-temperature performance of rejuvenated asphalt binders is evaluated using the DSR 4 mm plate low-temperature test. The low-temperature test results of the 4 mm plate of the DSR are consistent with those of the bending beam rheometer test. This experimental method has the advantages of a simple experimental operation, a low sample size, and a short experimental period. Therefore, this method conducts low-temperature performance tests on an asphalt binder scale [42,43,44]. Two parameters are measured as follows: the stiffness modulus (S) of the asphalt binder, which is used to evaluate the deformation resistance of the asphalt binder at low temperatures, and the creep rate (m), which is used to evaluate the rate of change in the creep modulus of the asphalt binder under the influence of low-temperature loading. A higher m value or a lower S value indicates that the asphalt binder has better low-temperature performance. The test temperatures are −6 °C, −12 °C, and −18 °C.

#### 3.1.4. Basic Properties of Rejuvenator Raw Materials

(1) Oil-like materials of aromatic hydrocarbons

Oil-like materials of aromatic hydrocarbons (Shijiazhuang, China) have many aromatic components; the addition of oil-like materials of aromatic hydrocarbons is mainly for the dispersion of dissolved asphaltine in the aging asphalt binder but also for the aging asphalt binder to supplement the reduction in aromatic components. An asphalt binder viscosity closer to the medium viscosity of oil-like materials of aromatic hydrocarbons was selected; its technical performance indicators are shown in Table 2. The testing method is based on the China Code specification [37].

(2) Plasticizer

Plasticizers (Shijiazhuang, China) can reduce the intermolecular forces in an asphalt binder and increase the mobility of the molecules. It can improve the flexibility and plasticity of materials [45,46]. The basic indicators are shown in Table 2.

(3) Modifier A

Modifier A (Jinan, China) can significantly improve the water stability of asphalt mixtures and enhance the interfacial bond between the asphalt and aggregate. The type I liquid modifier A produced in China was used in this study. The technical indicators are shown in Table 2.

### 3.2. Asphalt Mixture Preparation Process and Test Methods

#### 3.2.1. Design of Asphalt Mixture Mass Ratio of Asphalt Binder and Aggregate

**Figure 1:**
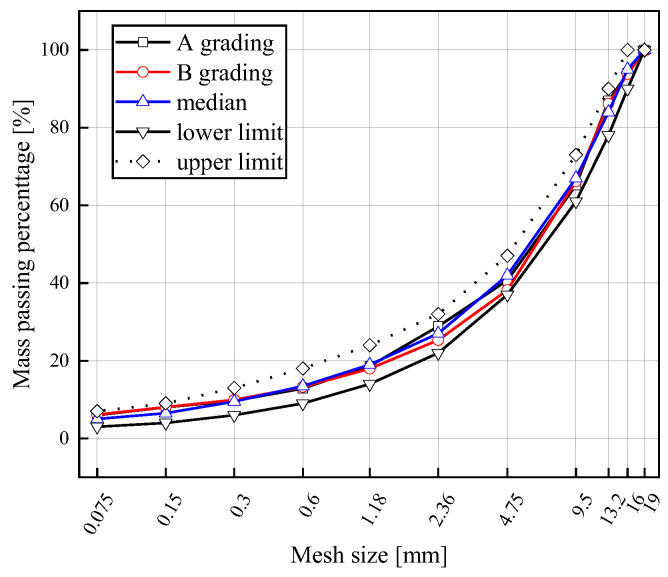
Grading curve. Note: Grade A is the grading of ordinary asphalt mixture, while Grade B is the grading of the rejuvenated asphalt mixture with 45% RAP.

The gradation type of the asphalt mixture used in this study is AC16 (the relevant indicators of AC16 meet the China Code [38]. Figure 1 is the grading screening curve. After the gradation screening and mass ratio of the asphalt binder and aggregate verification test, the optimum mass ratio of the asphalt binder and aggregate was finally determined to be 4.5, and the design porosity was 4%. The coarse aggregate used in the test was limestone, the fine aggregate was mechanism sand of limestone, and the filler was mineral powder of limestone.

#### 3.2.2. Asphalt Mixture Preparation Process

(1) Ordinary asphalt mixture

Coarse and fine aggregates are pre-mixed for 90 s. The asphalt binder is added and the mixing is continued for 90 s. Finally, a small amount of mineral powder is added and the mixing is continued for another 90 s. The ordinary asphalt mixture is produced.

(2) Rejuvenated asphalt mixture

Firstly, the new aggregates and 45% of the RAP are poured into the mixing pot and stirred for 90 s, then 7.82% (according to the proportion of aged asphalt binder in the RAP) of the rejuvenator is added, and is then stirred for another 90 s. Note that the content of the asphalt binder in RAP in this study was measured by the extraction method using a mixture analyzer(infraTest, Germany). According to the optimal oil and stone ratio, the new asphalt binder is added and is then stirred for another 90 s. Finally, a small amount of mineral powder is added and stirred for 90 s; the rejuvenated asphalt mixtures are made. There are three types of rejuvenators used in the validation tests of the mixture scale, rejuvenators C, H, and I. In other words, there are three types of rejuvenated asphalt mixtures.

#### 3.2.3. Test Methods for Road Performance of Asphalt Mixtures

All road performance test methods are conducted using the China Code [37].

(1) Water stability test

The water stability of each rejuvenated asphalt mixture is evaluated by performing an immersion Marshall stability test and a freeze–thaw split test.

Residual stability (RS) is one of the indicators for evaluating the water stability of asphalt mixtures. The larger RS value indicates that the water stability of the asphalt mixture is better. Eight standard Marshall specimens are prepared for each kind of asphalt mixture (75 times of compacting). The specimens were divided into two groups: the first group was placed in a 60 °C water bath box for 48 h, and the second group was immersed in a 60 °C water bath for 30 min; the stability of the two groups of specimens was tested using an automatic Marshall tester (Beijing Chaoyang Road Da Instrument Factory, Beijing, China), and the RS is calculated by Equation (4).

(4) $$RS=\frac{MS_{1}}{MS}\times 100\%$$

where *RS* is the residual stability, %; *MS*_1_ is the stability at 48 h of immersion, kN; MS is the stability at 30 min of immersion, kN.

The freeze–thaw splitting strength ratio (TSR) is another indicator for evaluating the water stability of asphalt mixtures. The larger the TSR value is, the better the water stability of asphalt mixtures. Each type of asphalt mixture is used to make eight standard Marshall specimens (50 times), using Equation (5) to calculate the TSR.

(5) $$TSR=\frac{R_{T2}}{R_{T1}}\times 100\%$$

where *TSR* is the freeze–thaw splitting strength ratio, %; *R*_T1_ is the splitting strength without the freeze–thaw cycle, MPa; *R*_T2_ is the splitting strength after the freeze–thaw cycle, MPa.

(2) High-temperature rutting test

A high-temperature rutting test is conducted to evaluate the high-temperature performance of each rejuvenated asphalt mixture; the specimen size is a 300 mm × 300 mm × 50 mm plate specimen. The test temperature is 60 °C, the wheel load is 0.7 MPa, the repeat wheel time is 60 min, the creep under different loading times is recorded automatically during the test, and the dynamic stability (DS) is used to evaluate the high-temperature performance of each asphalt mixture, and the greater the value of the DS is, the better the rutting performance of the asphalt mixture. The number of high-temperature rutting test specimens for each mixture is 3, and the test results are averaged.

(3) Low-temperature beam test

The low-temperature performance of each rejuvenated asphalt mixture is evaluated by the low-temperature beam test; the number of specimens of each asphalt mixture is 4, the specimen size is 50 mm × 30 mm × 35 mm, the span is 200 mm, the test temperature is −10 °C, and the loading rate is 50 mm/min. The maximum bending and tensile strains of the asphalt mixtures are used as the evaluation indexes for the low-temperature cracking resistance of the asphalt mixtures. The larger the maximum bending strain value of the asphalt mixture is, the better its low-temperature flexibility and crack resistance.

### 3.3. A Block Diagram Containing the Experimental Research Program

**Figure 2:**
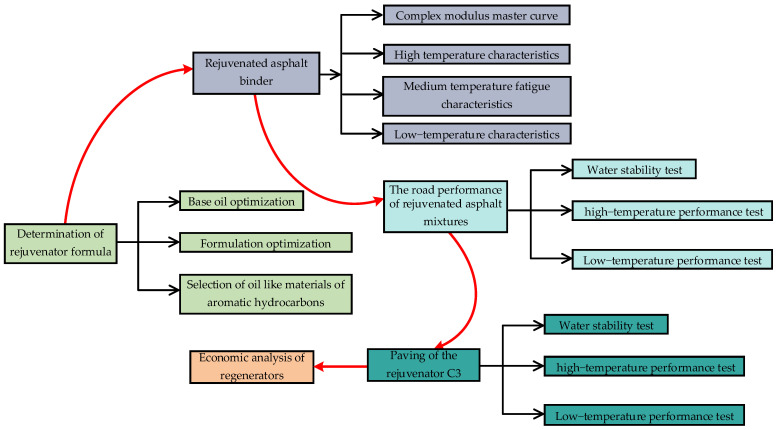
Technical roadmap.

The technical roadmap of this study is shown in Figure 2, which mainly includes the determination of the rejuvenator formula, verification tests of asphalt binders and asphalt mixtures at the scale, practical application of rejuvenators, and economic analysis.

## 4. Experimental Results and Discussion

### 4.1. Determination of Rejuvenator Formula

The previous study determined the optimal formulation of duck oil rejuvenator based on the star point effect surface method [47]. Roast duck oil, plasticizer, and modifier A are the main components of used bio-oil rejuvenators. Roast duck oil is a waste oil, and converting it into a rejuvenator can achieve waste utilization, reduce environmental pollution, and comply with sustainable development. Using the central composite design (CCD) for the experimental design requires the determination of their reasonable dosages. To determine the appropriate dosage of roasted duck oil, plasticizer, and modifier A, the efficient restoration of the properties of the aged asphalt binder by a single material was investigated. Different doses of waste bio-oil (1–9%) and plasticizer (1–5%) were mixed into the aged asphalt binder. The rejuvenated asphalt binder was tested by the penetration test, softening point test, and ductility test to analyze the reasonable dosage of each component of the rejuvenator. The manufacturer’s recommended dosage of modifier A (0.1–0.5%) had little effect on the indexes of the rejuvenated asphalt binder. Based on the CCD and response surface methodology (RSM), the optimum formulation of the rejuvenator for roasted duck oil was determined, and the above is the specific procedure. Due to the unstable performance of duck oil as a raw material and its small yield, it cannot meet the engineering needs of the mass production of rejuvenators. Therefore, there is an urgent need to find a base oil with a light component to replace duck oil as a raw material. Based on this, the first step of this paper is to optimize the base oil.

Base oil is one of three types: vacuum third-line oil, vacuum fifth-line oil, and oil-like materials of aromatic hydrocarbons; two materials, a plasticizer, and modifier A are added to form a rejuvenator.

Table A1 shows the kinds of asphalt binders, while Table A2 shows the composition of different types of rejuvenators. Table A1 and Table A2 are detailed in Appendix A.

The purpose of testing different formulations of rejuvenators is to determine the type of each material in the rejuvenator and the optimum mixing ratio. This process is designed to ensure the regeneration effect while simultaneously reducing the cost of the rejuvenator.

#### 4.1.1. Base Oil Optimization

(1) Complex modulus master curve

**Figure 3:**
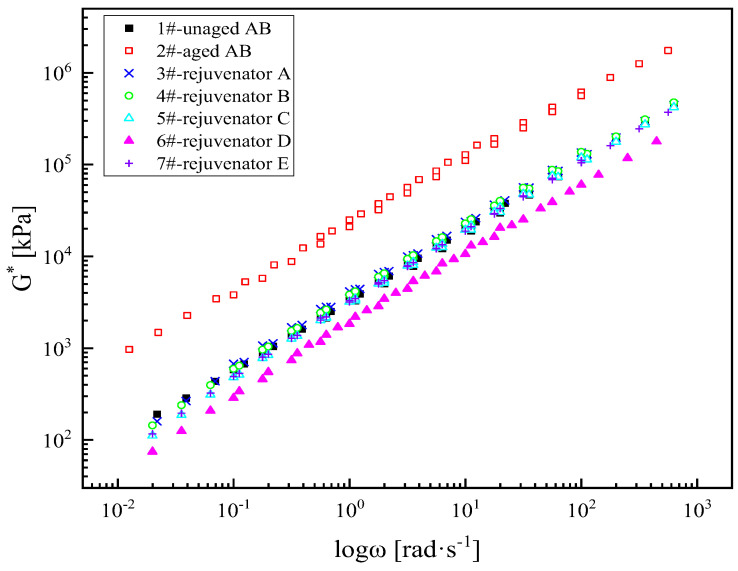
Complex modulus master curve of different types of rejuvenated asphalt binders.

The complex modulus of the SBS-modified asphalt binder after aging also has a relatively prominent elevation in the entire frequency range (Figure 3). At low frequencies, the modulus of the SBS-modified asphalt binder increases after aging, and its rutting resistance increases. However, at high frequencies, the modulus of the SBS-modified asphalt binder is higher than that of the unaged asphalt binder, and its low-temperature cracking resistance deteriorates. The complex modulus master curves of the rejuvenated asphalt binder after adding rejuvenators A, B, C, and E almost overlapped with those of the unaged asphalt binder. Compared with the virgin asphalt binder, the aged asphalt binder after the regeneration of rejuvenator D still has a certain degree of decrease in the full frequency range, which indicates that rejuvenator D has the best modulus restoration effect on the SBS-modified asphalt binder.

(2) High-temperature characteristics

**Figure 4:**
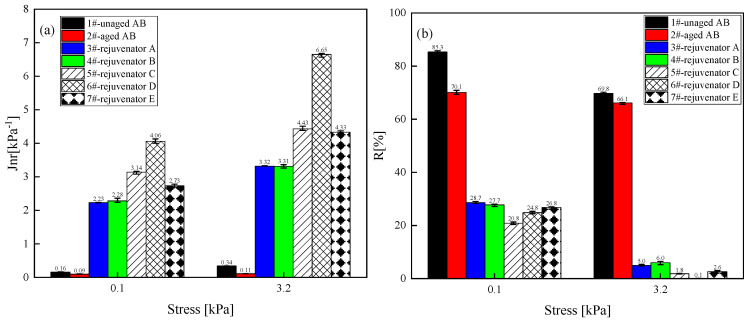
Unrecoverable flexibility and creep recovery of different types of rejuvenated asphalt binder at two stress levels. (**a**) Unrecoverable flexibility; (**b**) Creep recovery. Note: the lower limit of the error bar represents the minimum value of the sample test, the upper limit of the error bar represents the maximum value of the sample test, and the data label is the average value of the experimental data; it is the same below.

Figure 4 shows the evaluated unrecoverable creep softness and recovery rate of different rejuvenated asphalt binder types at two stresses (0.1 and 3.2 kPa) and 64 °C. The decrease in the irrecoverable creep softness of the aged SBS-modified asphalt binder indicates that the high-temperature performance of the asphalt binder has been improved, and the reduction in the creep recovery rate demonstrates that the high-temperature rutting performance of the aged asphalt binder has deteriorated. The Jnr value of the aged asphalt binder increased significantly after the addition of the rejuvenator. At the same time the R-value decreased significantly, indicating that the rejuvenator has a significant adverse effect on the high-temperature rutting performance of the aged asphalt binder. Moreover, the effect of rejuvenator D is also the most obvious. The closest effect of rejuvenator D is rejuvenator C, which can be used to reduce the dosage of the rejuvenator under the premise of guaranteeing the rejuvenator effect to reduce the construction cost. Furthermore, the negative impact of rejuvenator C on the high-temperature performance of asphalt is relatively minor. Therefore, taking the unrecoverable creep flexibility and creep recovery rate as the evaluation indexes, oil-like materials of aromatic hydrocarbons are preferred as the base oil of the rejuvenator from the perspective of regeneration efficiency and cost.

(3) Medium-temperature fatigue characteristics

**Figure 5:**
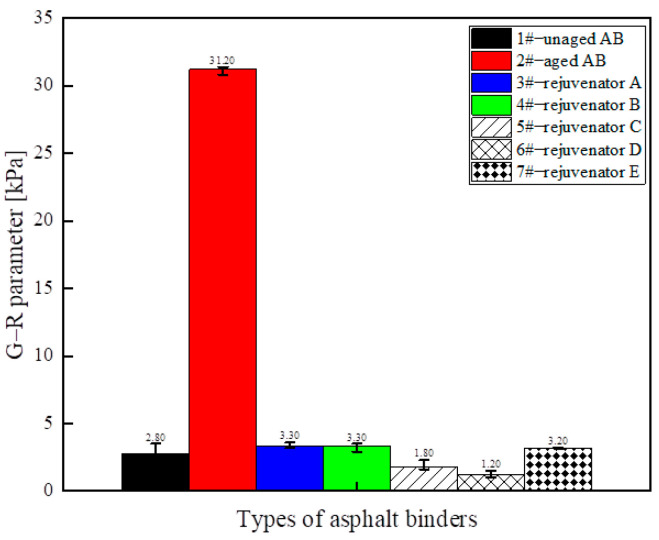
G–R parameter values for different types of rejuvenated asphalt binders.

The G–R parameter values of different rejuvenated asphalt binder types are shown in Figure 5. The G–R parameter reflects the cracking resistance of the asphalt binder under low-frequency conditions (or after aging). A higher G–R value indicates a higher elastic component and greater stiffness in the material, making it harder and more brittle. The G–R parameter can be used to evaluate the risk of cracking in aged asphalt; aging increases the G–R value, which is why the G–R value of the 2# aged asphalt binder is the highest. Adding a rejuvenator reduces the G–R value of the asphalt binder, thereby lowering the risk of cracking. The larger the value of the G–R parameter of the asphalt binder after aging, the worse the fatigue resistance. The G–R parameter value of the asphalt binder is significantly decreased by adding a rejuvenator, which can restore the medium-temperature fatigue resistance of the asphalt binder. Regenerator D showed the most significant improvement, and regenerator D was closest in effect to regenerator C. Under the premise of ensuring the regeneration effect, it is feasible to reduce the amount of rejuvenators to reduce the construction cost. Therefore, from the perspective of regeneration efficiency and cost, oil-like materials of aromatic hydrocarbons are preferred as the base oil of the rejuvenator, which is consistent with the conclusion obtained from the analysis of the results of the MSCR high-temperature test.

(4) Low-temperature characteristics

**Figure 6:**
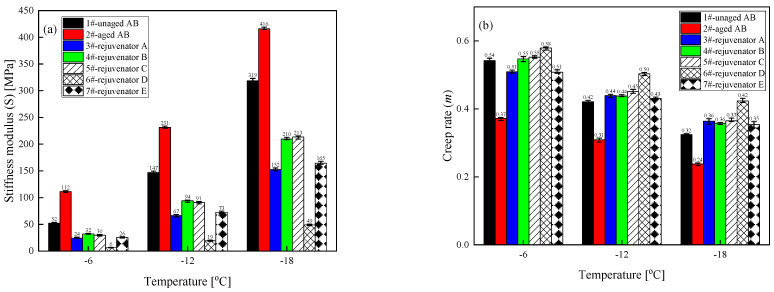
Values of stiffness modulus (S) and creep rate (m) for different types of rejuvenated asphalt binders. (**a**) Stiffness modulus (**b**) Creep rate.

The stiffness modulus (S) is the stress generated per unit strain in the asphalt binder at a specific time (typically 60 s), representing the material’s stiffness under unit strain. Figure 6 illustrates that at the three test temperatures, the aging process results in an increase in the stiffness modulus S value of the asphalt binder, a decrease in the creep rate m value, and a deterioration in the low-temperature performance of the asphalt binder. The addition of a rejuvenator will reduce the S value of the aged asphalt binder and increase its m value to different degrees; in other words, it improves flexibility and reduces the chances of cracking the aged asphalt binder at low temperatures. Among them, the improvement effect of rejuvenator D is the most obvious, and the recovery rate of the rejuvenators are all more than 100% (S and m); the regeneration effect of rejuvenator C remains second only to that of rejuvenator D.

#### 4.1.2. Formulation Optimization

To reduce the cost of the rejuvenator, the dosage of each component of the rejuvenator was reduced while ensuring the recovery effect of the rejuvenator, and the performance verification test was performed to finally determine the optimal formulation of the rejuvenator.

(1) Complex modulus master curve

**Figure 7:**
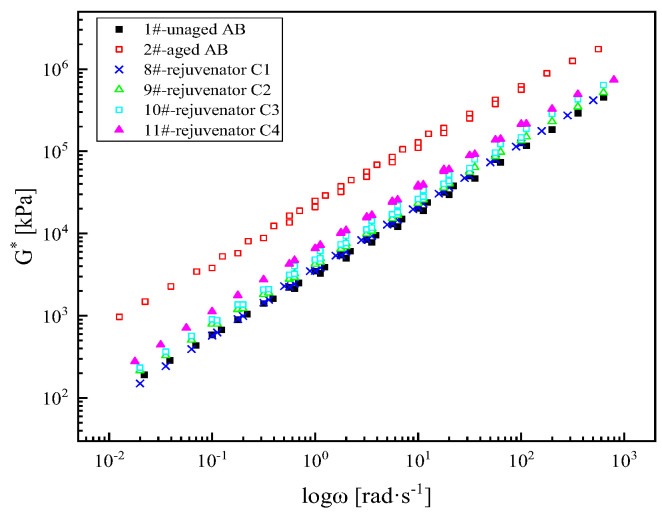
Complex modulus master curves of asphalt binder rejuvenated with different formulations of rejuvenator C.

As shown in Figure 7, the same raw materials and different formulations of rejuvenator C1, C2, C3, and C4 lead to a relatively significant increase in the complex modulus of the aged SBS (Styrene-Butadiene-Styrene)-modified asphalt binder in the whole frequency range as well. Rejuvenator C1 has the best rejuvenator effect. The rejuvenation effect order is rejuvenator C1, C2, C3, and C4. Reducing the base oil in the raw material or plasticizer and modifier A, the rejuvenation effect of the rejuvenator has a certain degree of decline, but after the optimization of the formulation of the rejuvenator, the recovery rate of the aged asphalt binder is still more than 90%; the rejuvenation effect is better.

(2) High-temperature characteristics

**Figure 8:**
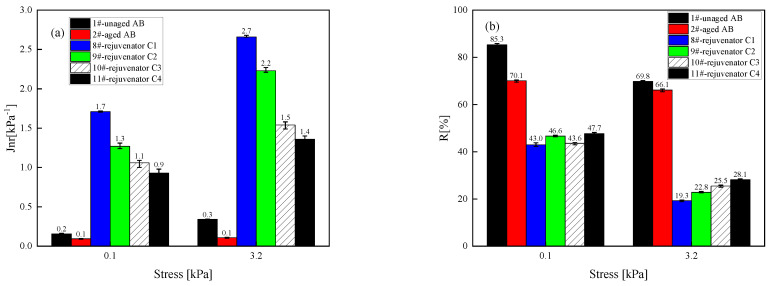
Unrecoverable flexibility and creep recovery of asphalt binder rejuvenated with different formulations of rejuvenator C at two stress levels. (**a**) Unrecoverable flexibility; (**b**) Creep recovery.

Figure 8 shows the evaluated unrecoverable creep flexibility and creep recovery rate of the asphalt binder rejuvenated with different formulations of rejuvenator C at two stresses and 64 °C. The high-temperature performance recovery effect of the rejuvenator on the aging asphalt binder becomes worse with the reduction in the content of the rejuvenator; in other words, the reduction in the content of any component of the rejuvenator reduces the Jnr value of the rejuvenated asphalt binder and increases its R-value, which means that the corresponding rejuvenated asphalt binder’s high-temperature rutting resistance becomes better.

(3) Medium-temperature fatigue characteristics

**Figure 9:**
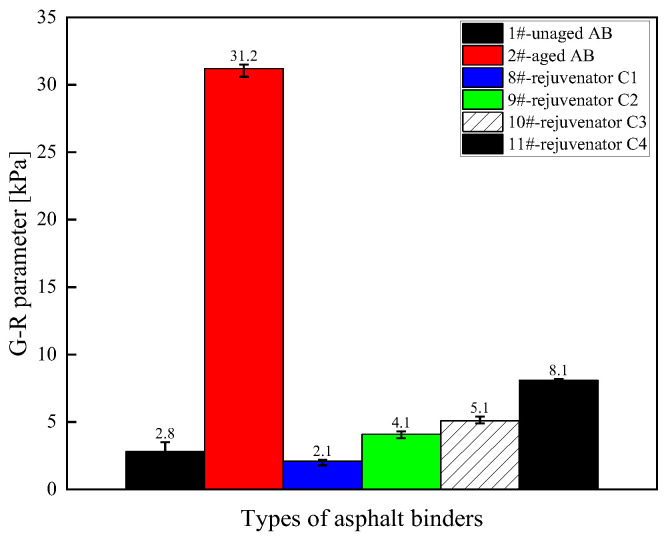
G–R parameter values of asphalt binder rejuvenated with different formulations of rejuvenator C.

Figure 9 shows the G–R parameter values of the asphalt binder rejuvenated with different formulations of rejuvenator C. The effect of the rejuvenator on the recovery of the medium-temperature fatigue properties of the aged asphalt binder decreases with the reduction in the content of each component of the rejuvenator. The G–R parameter of the rejuvenated asphalt binder increases when the content of any component of the rejuvenator decreases. In other words, the more the rejuvenator components are mixed, the better the fatigue properties of the rejuvenated asphalt binder are for the recycling effect.

(4) Low-temperature characteristics

**Figure 10:**
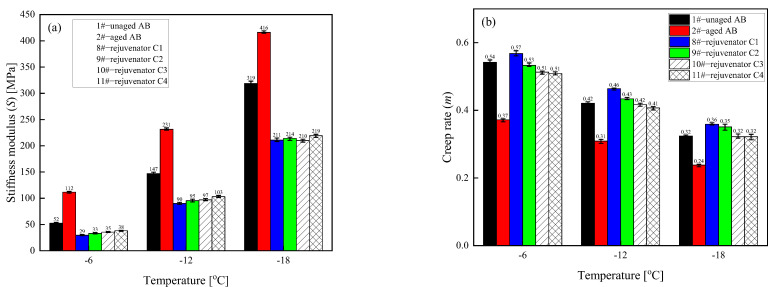
Values of modulus of stiffness (*S*) and creep rate (*m*) of asphalt binder rejuvenated with different formulations of rejuvenator C. (**a**) Modulus of stiffness; (**b**) Creep rate.

In Figure 10, the addition of different formulations of rejuvenator C reduces the *S* value of the aged asphalt binder. It increases the m value of the aged asphalt binder to different degrees under the three test temperatures, which indicates that adding the rejuvenator improves the low-temperature properties of the aged asphalt binder. The *S* value was the evaluation index, and the recovery rate of each rejuvenator for the low-temperature performance of the aged asphalt binder was more than 100%. The recovery effect of rejuvenator C on the low-temperature properties of the aged asphalt binder decreases with the reduction in the content of each component of the rejuvenator.

In summary, the reduction in the content of each component of the rejuvenator leads to the reduction in the improvement effect of the properties of the aged asphalt binder. However, the improvement effect of different formulations of rejuvenator C on the high-temperature properties of the aged asphalt binder is enhanced with the reduction in the dosage of each component of the rejuvenator. The rejuvenator components content is reduced, so comprehensively considering the cost of the engineering applications of the rejuvenator and regeneration effect, rejuvenator C3 is preferred for the best formula. Rejuvenator C3 accounted for 7.82% of the mass of the asphalt binder, 6% of which was oil-like materials of aromatic hydrocarbons, while plasticizer accounted for 1.52% and modifier A accounted for 0.3%.

#### 4.1.3. Selection of Oil-like Materials of Aromatic Hydrocarbons

**Figure 11:**
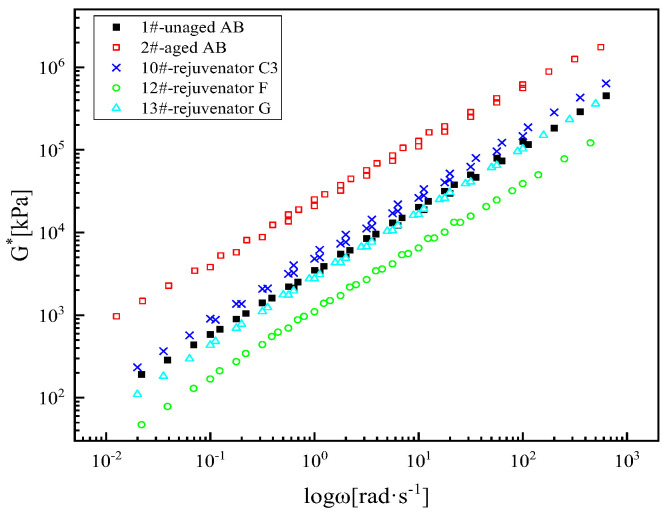
Complex modulus master curves of asphalt binders rejuvenated with the same formulation and different oil-like materials of aromatic hydrocarbons of rejuvenators.

**Figure 12:**
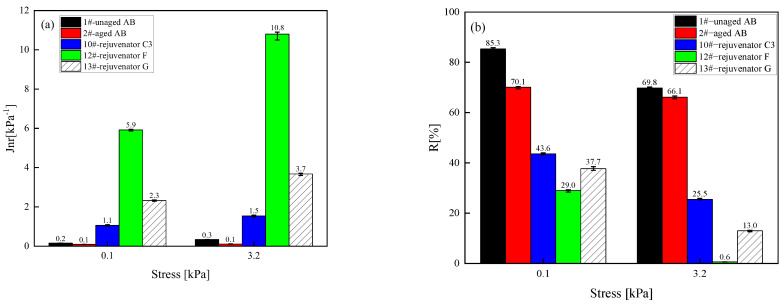
Unrecoverable flexibility and creep recovery of asphalt binder rejuvenated with the same formulation but different oil-like materials of aromatic hydrocarbons at two stress levels. (**a**) Unrecoverable flexibility; (**b**) Creep recovery.

**Figure 13:**
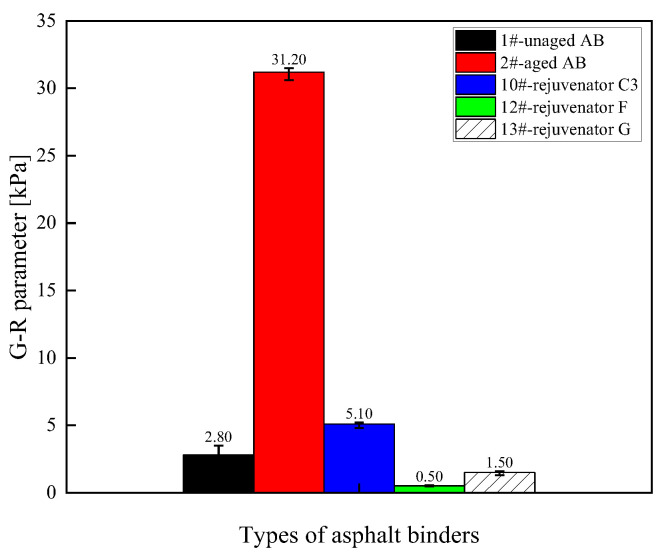
G–R parameter values for different types of rejuvenated asphalt binders.

**Figure 14:**
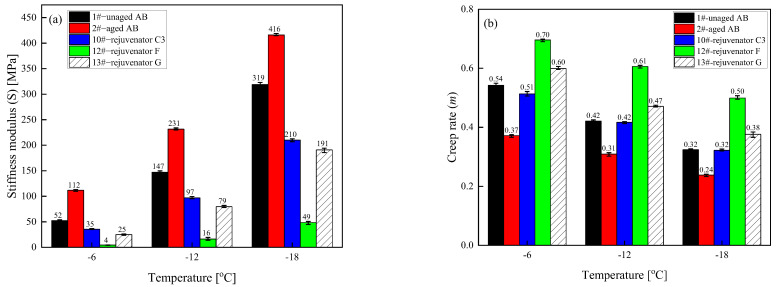
Values of stiffness modulus S and creep rate m for different types of rejuvenated asphalt binders. (**a**) Stiffness modulus; (**b**) Creep rate.

In Figure 11, for the complex modulus master curve, the three rejuvenators are ranked from good to bad in terms of recovery effect as rejuvenators G, F, and C3. Figure 12 shows the evaluated Jnr value and R-value of the asphalt binder rejuvenated by the rejuvenators with the same formulation but different oil-like materials of aromatic hydrocarbons at two stresses and 64 °C. Taking the Jnr value and R-value as the evaluation indexes, the aged asphalt binder rejuvenated by rejuvenator C3 has the best high-temperature properties. Figure 13 shows the G–R parameter values of the asphalt binder rejuvenated by the rejuvenators with the same formula but different oil-like materials of aromatic hydrocarbons, and the rejuvenator effects are ranked as follows: rejuvenator F, G, and C3. In Figure 14, the three formulations of rejuvenators enhance the low-temperature performance of the aged asphalt binder significantly, and the improvement effects are ranked in order as follows: rejuvenators F, G, and C3. The recovery rate of the *S* value and *m* value of the aged asphalt binder at three test temperatures is above 97%, and the regenerate effect has reached the standard. In order to ensure the high-temperature performance of recycled asphalt binder, it is more appropriate to choose rejuvenator C3. In conclusion, considering the high and low-temperature performance of the aged asphalt binder after rejuvenator, rejuvenator C3 is still confirmed to be the best formulation, and 1# oil-like materials of aromatic hydrocarbons are preferred as the raw material of the base oil for the rejuvenator.

In addition, the recovery rate of different rejuvenators for different performance indexes of aged asphalt binder is calculated as Equation (6):

(6) $$\mathrm{Recovery}\;\mathrm{rate}\;\mathrm{for}\;\mathrm{indicators}=\frac{\mathrm{Test}\;\mathrm{values}\;\mathrm{of}\;\mathrm{recycled}\;\mathrm{AB}-\mathrm{Test}\;\mathrm{values}\;\mathrm{of}\;\mathrm{aged}\;\mathrm{AB}}{\mathrm{Test}\;\mathrm{values}\;\mathrm{of}\;\mathrm{unaged}\;\mathrm{AB}-\mathrm{Test}\;\mathrm{values}\;\mathrm{of}\;\mathrm{aged}\;\mathrm{AB}}\times 100\%$$

Note that AB is the asphalt binder.

The recovery rate of representative evaluation indicators for the high-temperature, medium, and low-temperature properties of aged asphalt binders after adding different rejuvenators is presented in Table 3.

**Figure 15:**
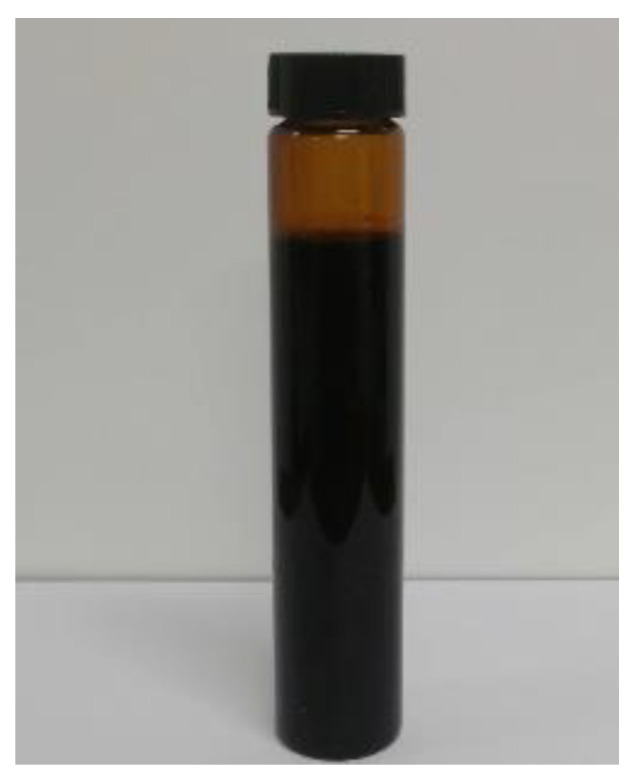
Liquid asphalt binder rejuvenator C3.

The indicators of the rejuvenated asphalt binder C3 are shown in Table 4. The aromatic content and density at 15 °C are measured values, and the other tested indicators comply with the technical requirements for asphalt binder RA-1 in the China Code [48]. The specific appearance of rejuvenator C3 is shown in Figure 15.

### 4.2. Study on the Effect of Different Rejuvenators on the Road Performance of Asphalt Mixtures

#### 4.2.1. Water Stability Test Results

**Figure 16:**
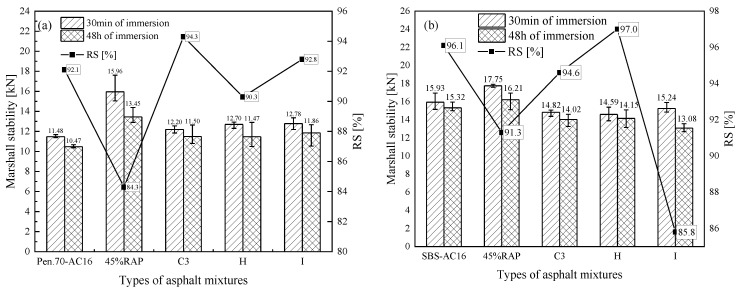
Comparison of the results of rejuvenated asphalt mixtures in the water-immersion Marshall test.

Figure 16a,b are the results of the immersion Marshall stability test of rejuvenated asphalt mixtures with the new asphalt binders of Pen.70 VAB and the SBS-modified asphalt binder, respectively. Taking the residual stability of immersion Marshall as the evaluation index, the residual stability of the mixtures was reduced with the addition of 45% RAP. The addition of rejuvenators C3 and H increased the residual stability of the mixtures. However, for rejuvenated asphalt mixtures where the new asphalt binder is the SBS-modified asphalt binder, the addition of rejuvenator I does not improve the residual stability of the asphalt mixture. In summary, the water stability of the asphalt mixtures deteriorated after the addition of RAP, and adding a rejuvenator could enhance the water stability of the asphalt mixtures. The effect of rejuvenator I on the enhancement of the stability performance of the mixture is relatively poor. However, the water stability of all rejuvenated asphalt mixtures meets the specification requirements.

**Figure 17:**
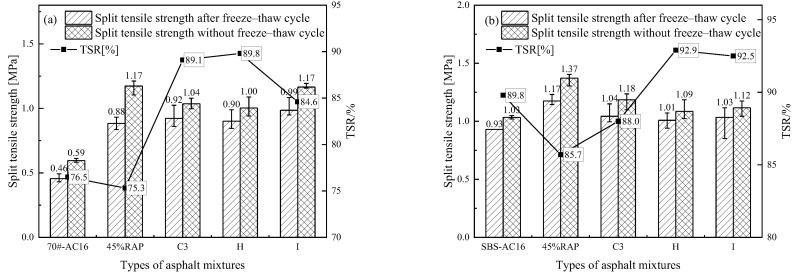
Comparison of freeze–thaw splitting test results for rejuvenated asphalt mixtures.

Figure 17a,b show the freeze–thaw splitting test results of the rejuvenated asphalt mixtures with the new asphalt binder of Pen.70 VAB and the SBS-modified asphalt binder, respectively. Taking the freeze–thaw splitting strength ratio (TSR) as the evaluation index, for Pen.70 VAB, the splitting tensile strength of the rejuvenated asphalt mixtures increased after adding 45% RAP. After adding the rejuvenator, the TSR of the rejuvenated asphalt mixture is reduced, that is, the water stability deteriorates, but the TSR meets the specification requirements of TSR > 75%. For the SBS-modified asphalt mixture, after adding 45% RAP, the split tensile strength of the rejuvenated asphalt mixture is reduced, and the TSR of the asphalt mixture after adding the rejuvenator increases, that is, the water stability is improved, and they all meet the specification requirements of TSR > 80% of the technical indicators. The addition of a rejuvenator for the rejuvenated asphalt mixture water stability effect law is not uniform, but the impact is not significant. The water stability of the rejuvenated asphalt mixture is good. In addition, the results of two stability tests indicate that the developed rejuvenator C3 has a more significant effect on the Pen.70 VAB.

#### 4.2.2. Analysis of High-Temperature Performance Test Results

**Figure 18:**
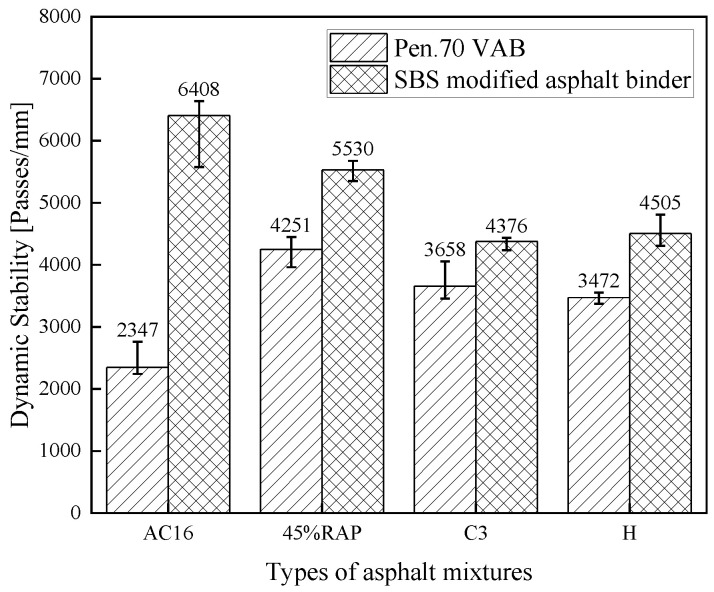
Comparison of dynamic stability test results for rejuvenated asphalt mixtures.

Figure 18 shows the high-temperature rutting test results of the rejuvenated asphalt mixtures with the new asphalt binder of Pen.70 VAB and the SBS-modified asphalt binder, respectively. The DS of the rejuvenated asphalt mixture with Pen.70 VAB as the new asphalt binder increases significantly after adding RAP, i.e., the high-temperature rutting resistance improves significantly, and the rejuvenated asphalt mixture with the SBS-modified asphalt binder showed a slight decrease in DS values compared to the unaged asphalt mixtures, i.e., the high-temperature properties deteriorate. The reason may be the aged asphalt binder in RAP is the VAB, which has weak interface bonding with the SBS-modified asphalt binder. After adding the rejuvenator, the dynamic stability of the rejuvenated asphalt mixture is reduced. In other words, the rejuvenator plays a softening effect, and the rejuvenated asphalt mixture’s high-temperature rutting resistance is reduced, but they meet the specification requirements: the virgin asphalt mixture’s DS > 1000 (passes/mm), and the SBS-modified asphalt mixture’s DS > 2800 (passes/mm). Moreover, the differences in the effects of the three rejuvenators on the high-temperature properties of the asphalt mixtures were minor.

#### 4.2.3. Low-Temperature Performance Test Results

**Figure 19:**
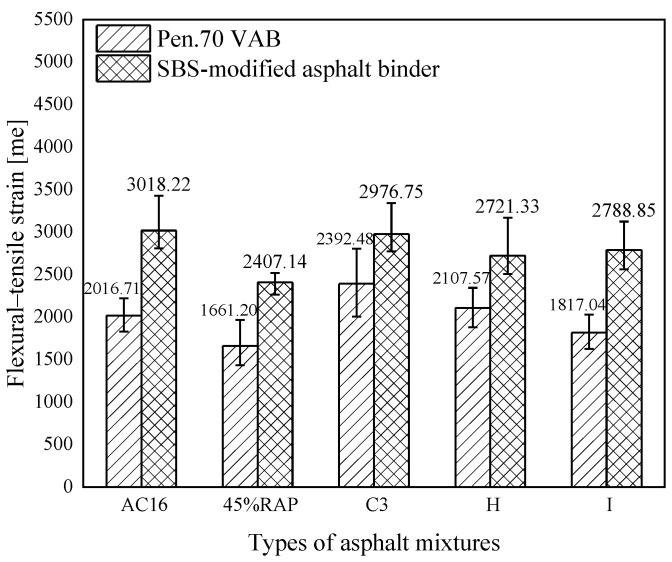
Comparison of flexural and tensile strain test results for rejuvenated asphalt mixtures.

In Figure 19, after adding RAP to the asphalt mixture, no matter whether the new asphalt binder is Pen.70 VAB or the SBS-modified asphalt binder, the bending and tensile strain of the rejuvenated asphalt mixture decreases, and the low-temperature cracking resistance becomes worse. Adding a rejuvenator can enhance the bending and tensile strains of the rejuvenated asphalt mixtures, that is, it can increase the low-temperature performance of asphalt mixtures. According to the test data, rejuvenator C3 for the rejuvenated asphalt mixture has a good low-temperature performance recovery effect. The bending strain of the asphalt mixture after adding 45% RAP is lower than the specification requirements, and the low-temperature performance of the rejuvenated asphalt mixture after adding 7.82% of rejuvenators C3 and H meets the standards. The results show the developed rejuvenator C3 for rejuvenated asphalt mixtures of Pen.70 VAB to improve the effect is more obvious; the low-temperature performance of the rejuvenated asphalt mixtures of Pen.70 VAB is better than an ordinary asphalt mixture. Rejuvenator I is poor in enhancing the low-temperature property of rejuvenated asphalt mixtures of Pen.70 VAB. Compared with the other two commercial rejuvenators, rejuvenator C3 was the best for improving the low-temperature performance of rejuvenated asphalt mixtures.

### 4.3. Paving of the Rejuvenator C3 Test Section

**Figure 20:**
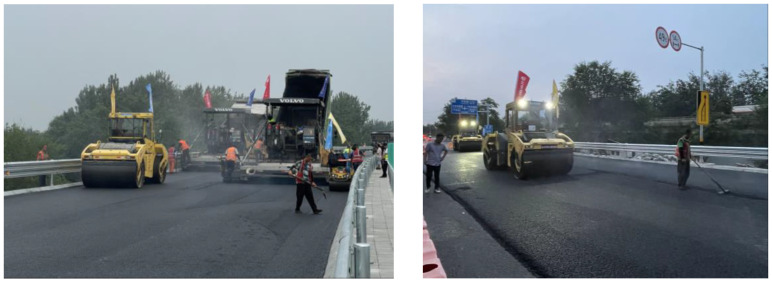
Experimental road paving site for rejuvenator.

To verify the practical application effectiveness of the rejuvenator, a test section was paved using the rejuvenator. Figure 20 shows the construction site. Project name: Huangliang Road (K7+200~K8+000.116) Reconstruction Project. Construction section: left carriageway from K7+200 to K8+000.116, where asphalt concrete surface course was paved. The gradation type used was SMA13. The dosage of RAP was 35%. The test section was 50 m in length and 10.75 m in width. The designed pavement thickness was 4 cm, and the measured thickness was 4.1 cm. The paving procedure included three passes of initial compaction, two passes of intermediate compaction, and two passes of final compaction. The compaction temperatures were as follows: initial compaction at 160–170 °C, intermediate compaction at 130–140 °C, and final compaction at approximately 110 °C.

In order to verify whether the basic road performance of the rejuvenator test section meets the specification requirements and to evaluate the rejuvenating effect of the agent, a comparative verification test was conducted. Four types of asphalt mixtures were subjected to high and low-temperature performance tests to assess the basic road performance of the mixtures used in the test section. The four types of mixtures were as follows:

(a) Control group (conventional SMA13 asphalt mixture);
(b) SMA13 asphalt mixture with a rejuvenator;
(c) Control group + 16 h aging;
(d) SMA13 asphalt mixture with a rejuvenator + 16 h aging.

The first two types of asphalt mixtures were taken directly from the mixing plant and molded in the laboratory, with a discharge temperature of 184 °C. The 16 h aged samples were aged under the following conditions.

Material was discharged at 1:00 a.m., with a temperature of 180 °C upon arrival at the construction site. Paving began at 4:00 a.m., and mixture samples were taken from the site and stored in insulated containers. After paving was completed at 9:00 a.m., the samples were brought back to the laboratory and kept in a 180 °C oven for 6 h before molding. The total time from discharge to molding was 16 h. This portion of the experiment was used to evaluate the durability of the rejuvenator through the road performance of aged asphalt mixtures. The detailed analysis of the test results is as follows.

Additionally, it is worth noting that during this 16 h aging process, the temperature of the asphalt mixture remains above the compaction temperature (180 °C). As a result, significant aging of the asphalt mixture may occur during this period. According to the China Code specification [37], the short-term aging is 135 °C aging for 4 h. (Specifically, the asphalt mixture is evenly spread in the enameled pan, with a loose thickness of about 21 kg/m^2^~22 kg/m^2^. The mixture is placed in an oven at 135 °C ± 3 °C and heated under forced ventilation for 4 h ± 5 min, and the mixture is turned over in the specimen tray with a shovel once an hour. After 4 h of heating, the mixture is removed from the oven and used for the test). Blankenship et al. found that loose asphalt mixtures aged for 24 h at 135 °C are equivalent to five years of in situ aging in the model [49]. In this study, the 16 h aging period is much more extended than the four hours of short-term aging, and the temperature is also higher than 135 °C. Therefore, it can be considered that this condition can, to some extent, verify the durability of the developed rejuvenator.

#### 4.3.1. Water Stability Test Results of Experimental Road

**Figure 21:**
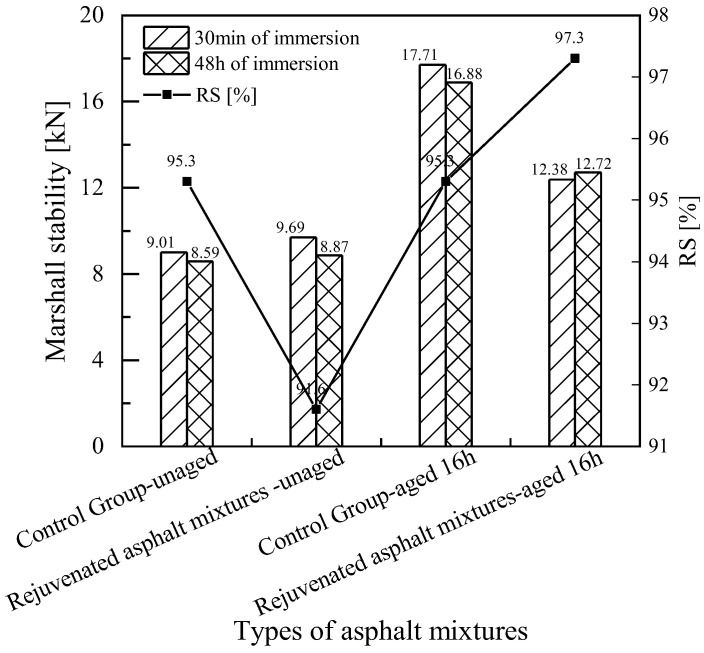
Comparison of Marshall test results for immersion of rejuvenated asphalt mixtures on experimental roads.

**Figure 22:**
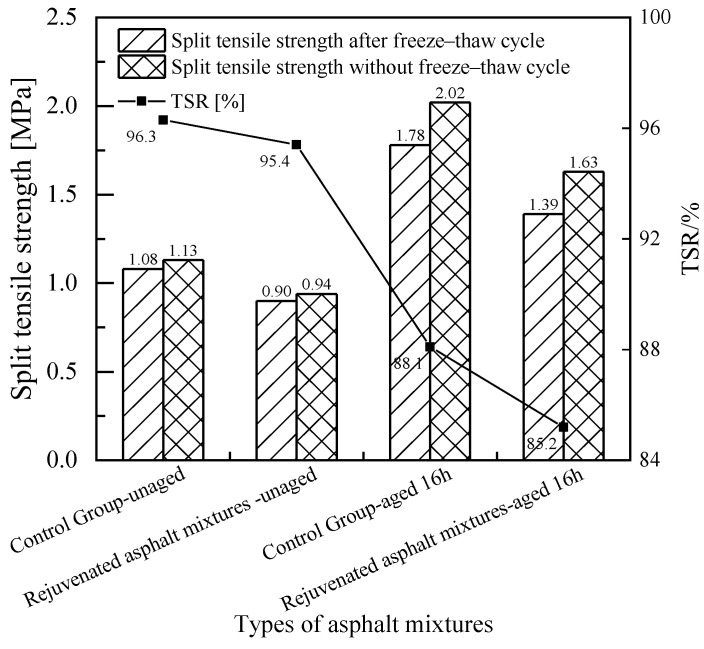
Comparison of freeze–thaw splitting test results of rejuvenated asphalt mixtures on test roads.

As shown in Figure 21, the residual stability of the asphalt mixtures is all above 90%, indicating good water stability. As shown in Figure 22, the tensile strength ratio (TSR) of the recycled asphalt mixtures is lower than that of the conventional asphalt mixtures. However, all values meet the technical requirement of TSR > 80% specified in the Specifications for Asphalt and Asphalt Mixture Testing in Highway Engineering.

In terms of splitting tensile strength, the strength growth rate of the asphalt mixture with the rejuvenator is significantly lower than that of the control group, indicating that the durability of the asphalt mixture improves after adding the rejuvenator. After 16 h of short-term aging, the residual stability still meets the specification requirement of greater than 80%.

#### 4.3.2. Analysis of High-Temperature Performance Test Results of Experimental Road

**Figure 23:**
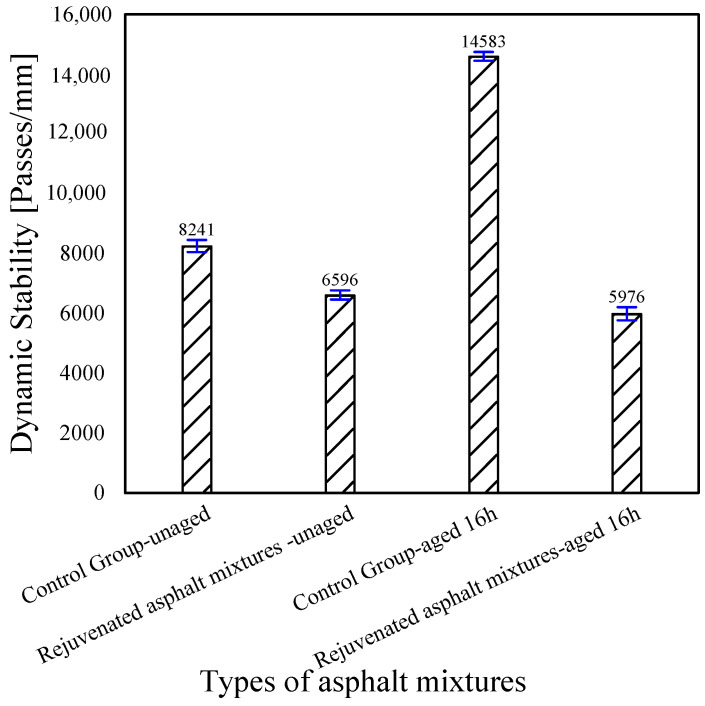
Comparison of dynamic stability test results of rejuvenated asphalt mixtures from the test section.

As shown in Figure 23, using dynamic stability (DS) as the evaluation index, the high-temperature performance of the asphalt mixture slightly decreased after the addition of the rejuvenator. After 16 h of short-term aging, the dynamic stability of the control group significantly increased—by approximately 77%—indicating a notable improvement in rutting resistance. This suggests that the asphalt mixture underwent significant aging and became harder. In contrast, the dynamic stability of the asphalt mixture with the rejuvenator remained nearly unchanged, indicating good durability of the rejuvenator.

All four asphalt mixtures met the high-temperature rutting performance requirement specified in the Test Specifications for Asphalt and Asphalt Mixtures in Highway Engineering: DS > 2800 (passes/mm) for SBS-modified asphalt mixtures.

#### 4.3.3. Low-Temperature Performance Test Results of Experimental Road

**Figure 24:**
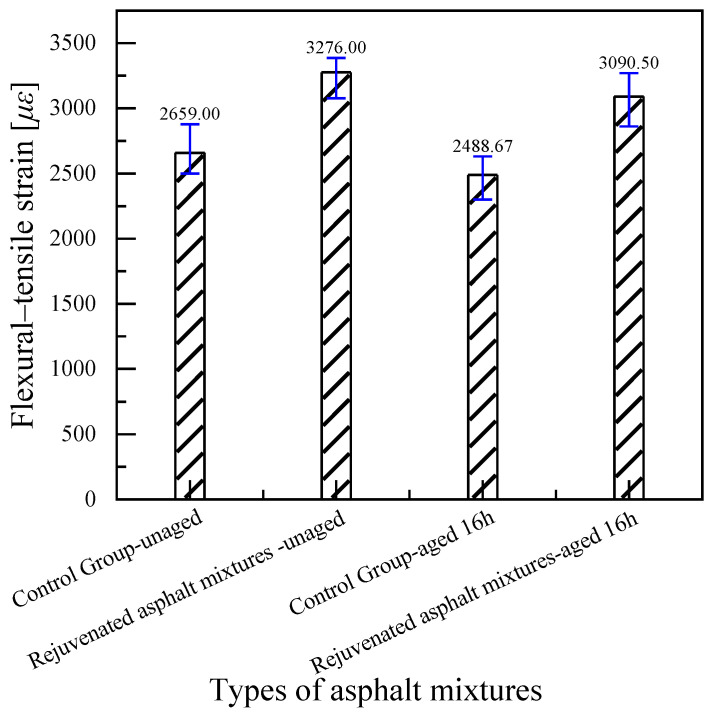
Comparison of flexural and tensile strain test results of rejuvenated asphalt mixtures on test roads.

As shown in Figure 24, using flexural strain as the evaluation indicator, the low-temperature performance of the asphalt mixture improved by 23% after the addition of the rejuvenator. After 16 h of short-term aging, the flexural strain of the rejuvenated asphalt mixture was 24% higher than that of the control group, indicating a significant rejuvenating effect of the additive.

### 4.4. Economic Analysis of Regenerators

In the process of highway maintenance, each kilometer of asphalt pavement (four lanes, milling three layers) produces about 0.7 million tons of RAP. The waste material is crushed and screened, and the rejuvenator is added for recycled asphalt pavement paving, which has significant economic benefits as follows.

(1) It can save aggregate resources; if recovering 7 thousand tons/km of old materials all for road repair, reconstruction, and new construction, it can save the same amount of new aggregate (CNY 80 per ton), and then, subtracting the cost of transporting old materials (CNY 10 per ton), the value of the recycled old materials is 0.7 × (80 − 10) = CNY/km 490 thousand CNY/km. (2) It can save the asphalt binder resources; if recovering 7 thousand tons/km of old material all for road repair, reconstruction, and new construction projects, the recycled asphalt mixture of the mass ratio of the asphalt binder and aggregate is about 5.0%, and the activation of aging asphalt efficiency by the rejuvenator is of more than 70%. The asphalt binder that can be saved is 0.7 × 5.0% × 70% = 0.245 thousand tons/km; the value is 0.0245 × 4000 = CNY/km 980 thousand (the price of asphalt binder is CNY 4000 per ton). In summary, the total cost of raw materials saved can be CNY/km 1 million 470 thousand.

Commodity rejuvenator H and I used in the market price is about CNY/ton 15 thousand CNY/ton, and the price for the independent research and development use of the high-performance rejuvenator C3 is CNY/ton 13 thousand. Recycled 0.7 million tons/km of old materials are all used for the regeneration of asphalt pavement; if 10% rejuvenator is added to the quality of aged asphalt binder mixing, the rejuvenator dosage is about 7000 × 5.0% × 10% = 35 tons/km. Compared with the traditional commercial rejuvenators, the self-developed high-performance rejuvenator C3 can save 35 × (1.5 − 1.3) = CNY/km 70 thousand. Simultaneously, rejuvenator C3 can enhance the durability of rejuvenated asphalt mixtures and significantly save maintenance costs.

## 5. Conclusions

This paper developed a liquid asphalt rejuvenator and optimized the formula. The regeneration effect of the rejuvenator on the RAP was verified from the two scales of the asphalt binder and asphalt mixture. The road performance verification test of rejuvenated asphalt mixtures with rejuvenators was carried out. A comparative analysis of performance and cost with two commonly used commercial rejuvenators was performed. The results are obtained as follows:

(1) Oil-like materials of aromatic hydrocarbons were selected as the lightweight softener through performance optimization. The formula of the rejuvenator was determined as rejuvenator C3, in which oil-like materials of aromatic hydrocarbons accounted for 6%, plasticizer accounted for 1.52%, and modifier A accounted for 0.3%; the total dosage was 7.82% of the mass of the asphalt binder, and the developed rejuvenator met the technical requirements of the Chinese specification (JTG/T 5521-2019) for the asphalt rejuvenator RA-1.

(2) From the verification test of the asphalt binder scale, adding 7.82% of rejuvenator C3 can significantly increase the low-temperature cracking resistance and medium-temperature fatigue performance of the aged asphalt binder, and the performance of the rejuvenated aged asphalt binder is almost the same as that of the virgin asphalt binder in low and medium temperatures, but there is an adverse effect on the high-temperature performance of the asphalt binder.

(3) The influence of the addition of rejuvenators on the water stability of rejuvenated asphalt mixtures is not uniform. However, the effect is small, and the water stability of the rejuvenated asphalt mixture is good. The rutting resistance of the asphalt mixture is improved after adding RAP. Adding rejuvenator C3 plays a softening effect, and the high-temperature rutting resistance of rejuvenated asphalt mixtures is deteriorating but still meets the specification requirements. The low-temperature performance of asphalt mixtures after the addition of RAP to reduce the low-temperature road performance does not meet the specification requirements; the addition of 7.82% of rejuvenator C3 significantly improves the low-temperature performance of rejuvenated asphalt mixtures. In addition, the engineering application of rejuvenator C3 on actual pavements has shown significant effectiveness.

(4) Rejuvenated asphalt mixtures with rejuvenator C3 have the best road performance compared to rejuvenator H and rejuvenator I. The cost of road maintenance with rejuvenator C3 is the lowest. This is also an advantage of the developed rejuvenator C3.

## Figures and Tables

**Table 1:** Basic properties of asphalt binders.

Test Items	Unit	Standard Value	Pen.70 Measured Value	SBS Measured Value
Penetration (25 °C, 5 s, 100 g)	0.1 mm	60~80	75	70
Ductility (10 °C is used for Pen.70 VAB, 5 °C is used for SBS-modified asphalt binder)	cm	binder	>100	38
Softening point	°C	46	48.0	75.5
Flash point	°C	260	263	278

**Table 2:** Basic properties of rejuvenator raw materials.

	Oil-like Materials of Aromatic Hydrocarbons	Plasticizer	Modifier A
Density (25 °C g·cm^−3^)	1.045	1.02	0.97
Kinematic viscosity (mm^2^/s 100 °C)	23	-	-
Flashpoint/°C	230	218	210
Boiling point/°C		386	-
Aromatics content/% (weight percentage)	86.3	-	-

**Table 3:** Recovery rate of each performance after adding different rejuvenators [%].

Performance	Index	A	B	C	D	E	C1	C2	C3	C4	F	G
Complex modulus	G* at 10 rad/s	96.19	97.20	100.93	110.73	101.59	99.62	102.21	93.38	81.46	115.26	104.11
High-temperature rutting properties	J_nr_ at 3.2 kPa	1384.05	1379.74	1862.50	2819.40	1819.40	1099.57	914.22	616.81	539.22	4608.19	1539.22
Medium-temperature fatigue performance	G–R parameter	98.24	98.24	103.52	105.63	98.59	102.46	95.42	91.90	81.34	108.10	104.58
Cracking resistance at low temperatures	−18 °C, m value	146.51	139.53	147.67	215.12	134.88	140.70	131.40	97.67	97.67	303.49	160.47
	−18 °C, S value	271.11	212.12	208.58	377.51	258.59	211.06	207.72	211.74	202.36	377.99	231.65

**Table 4:** Indicators of rejuvenator C3.

Number	Test Items	Unit	Technical Indicators	Test Results	Result Determination
1	Viscosity (60 °C)	mm^2^/s	50–175	96.43	Qualified
2	Flash point	°C	≥220	230	Qualified
3	Content of saturated fragrance	%	≤30	12.45	Qualified
4	Content of sweet fragrance	%	Actual measurement records	59.73	Measured value
5	Viscosity ratio before and after the thin film oven test	--	≤3	0.55	Qualified
6	Mass changes before and after the thin film oven test	%	≤4, ≥−4	0.85	Qualified
7	Density (15 °C)	g/cm^3^	Actual measurement records	1.06	Measured value

## Data Availability

The original contributions presented in the study are included in the article, further inquiries can be directed to the corresponding author.

## Appendix A

**Table A1:** Types of asphalt binder samples.

Sample Number	Sample Name
1#	Unaged SBS-modified asphalt binder
2#	Aged SBS-modified asphalt binder
3#	Aged SBS-modified asphalt binder + Rejuvenator A
4#	Aged SBS-modified asphalt binder + Rejuvenator B
5#	Aged SBS-modified asphalt binder + Rejuvenator C
6#	Aged SBS-modified asphalt binder + Rejuvenator D
7#	Aged SBS-modified asphalt binder + Rejuvenator E
8#	Aged SBS-modified asphalt binder + Rejuvenator C1
9#	Aged SBS-modified asphalt binder + Rejuvenator C2
10#	Aged SBS-modified asphalt binder + Rejuvenator C3
11#	Aged SBS-modified asphalt binder + Rejuvenator C4
12#	Aged SBS-modified asphalt binder + Rejuvenator F
13#	Aged SBS-modified asphalt binder + Rejuvenator G

**Table A2:** Types and formulas of rejuvenators.

The Type of Rejuvenator and Its Proportion to the Mass Fraction of the Asphalt Binder	Base Oil Type and Content	Plasticizer Content	Proportion of Modifier A
Rejuvenator A, 11.72%	Oil of the third vacuum stream oil 9%	2.28%	0.44%
Rejuvenator B, 11.72%	Oil of the fifth vacuum stream oil 9%	2.28%	0.44%
Rejuvenator C, 11.72%	1# oil-like materials of aromatic hydrocarbons 9%	2.28%	0.44%
Rejuvenator D, 11.72%	Commodity rejuvenator I		
Rejuvenator E, 11.72%	Roast duck oil 9%	2.28%	0.44%
Rejuvenator C1, 8.72%	1# oil-like materials of aromatic hydrocarbons 6%	2.28%	0.44%
Rejuvenator C2, 7.22%	1# oil-like materials of aromatic hydrocarbons 4.5%	2.28%	0.44%
Rejuvenator C3, 7.82%	1# oil-like materials of aromatic hydrocarbons 6%	1.52%	0.3%
Rejuvenator C4, 6.32%	1# oil-like materials of aromatic hydrocarbons 4.5%	1.52%	0.3%
Rejuvenator F, 7.82%	2# oil-like materials of aromatic hydrocarbons 6%	1.52%	0.3%
Rejuvenator G, 7.82%	3# oil-like materials of aromatic hydrocarbons 6%	1.52%	0.3%
Rejuvenator H, 7.82%	Commodity rejuvenator II		
Rejuvenator I, 7.82%	Commodity rejuvenator III		

Notes: A to E are comparative analyses of rejuvenators from different raw materials. C1–C4 are used to determine the optimal ratio of the rejuvenator. F and G are comparative analyses of aromatic hydrocarbon oily substances from different manufacturers. H and I are two types of commercial rejuvenators in order to compare their performance with the developed rejuvenator.

## References

[1] Mohd Hasan M.R., You Z. (2015). Estimation of cumulative energy demand and green house gas emissions of ethanol foamed WMA using life cycle assessment analysis. Constr. Build. Mater..

[2] Guo M., Liang M., Du X. (2023). Evaluation on Feasibility of Carbon Black and Hindered Amine Light Stabilizer as UV-Resistant Additives of Asphalt Binder. J. Test. Eval..

[3] Guo M., Yin X., Du X., Tan Y. (2023). Effect of aging, testing temperature and relative humidity on adhesion between asphalt binder and mineral aggregate. Constr. Build. Mater..

[4] Cong P., Luo W., Xu P., Zhao H. (2015). Investigation on recycling of SBS modified asphalt binders containing fresh asphalt and rejuvenating agents. Constr. Build. Mater..

[5] Chen A., Liu G., Zhao Y., Li J., Pan Y., Zhou J. (2018). Research on the aging and rejuvenation mechanisms of asphalt using atomic force microscopy. Constr. Build. Mater..

[6] Guo N., You Z., Zhao Y., Tan Y. (2015). Performance of Warm Mix Asphalt with Recycled Asphalt Mixtures Considering the Effect of Rejuvenating Agent. J. Build. Mater..

[7] Xiao F., Su N., Yao S., Amirkhanian S., Wang J. (2019). Performance grades, environmental and economic investigations of reclaimed asphalt pavement materials. J. Clean. Prod..

[8] Roy S., Hossain Z., Baumgardner G., Zaman M. (2023). Micro-mechanistic and Spectroscopic Analysis of RAP-Blended Asphalt Binders Rejuvenated with Waste Oils. Int. J. Pavement Res. Technol..

[9] Cao W., Wang C. (2019). Fatigue performance characterization and prediction of asphalt binders using the linear amplitude sweep based viscoelastic continuum damage approach. Int. J. Fatigue.

[10] Ma W., Huang T., Guo S., Yang C., Ding Y., Hu C. (2019). Atomic force microscope study of the aging/rejuvenating effect on asphalt morphology and adhesion performance. Constr. Build. Mater..

[11] Wang Z., Ding H., Ma X., Yang W., Ma X. (2024). Predicting Dynamic Properties and Fatigue Performance of Aged and Regenerated Asphalt Using Time–Temperature–Aging and Time–Temperature–Regenerator Superposition Principles. Coatings.

[12] Jing W., Ding S., Wang L., Lu W., Ge D. (2023). Performance evaluation of styreneic methyl copolymer regenerated SBS-modified asphalt and its mixture with high content RAP. Case Stud. Constr. Mat..

[13] Mogawer W., Bennert T., Daniel J.S., Bonaquist R., Austerman A., Booshehrian A. (2012). Performance characteristics of plant produced high RAP mixtures. Road Mater. Pavement Des..

[14] Ma H., Guo F., Han J., Zhi P. (2024). Analysis of Rheological Properties and Regeneration Mechanism of Recycled Styrene–Butadiene–Styrene Block Copolymer (SBS) Modified Asphalt Binder Using Different Rejuvenators. Materials.

[15] Zhang R., You Z., Ji J., Shi Q., Suo Z. (2021). A Review of Characteristics of Bio-Oils and Their Utilization as Additives of Asphalts. Molecules.

[16] Qin Y., Dong R., Li Y. (2024). Characterization of light components in rubber-oil mixtures reclaimed asphalt and their effect on the self-healing performance of asphalt. Constr. Build. Mater..

[17] Xu S., Sun D., Zhang X. (2024). Study of Aging Asphalt Recovery Using Peanut Oil Residue as a Biomass Regenerator. J. Transp. Eng. Part B Pavements.

[18] Guo M., Liang M., Sreeram A., Bhasin A., Luo D. (2022). Characterisation of rejuvenation of various modified asphalt binders based on simplified chromatographic techniques. Int. J. Pavement Eng..

[19] Guo M., Liu H., Jiao Y., Mo L., Tan Y., Wang D., Liang M. (2020). Effect of WMA-RAP technology on pavement performance of asphalt mixture: A state-of-the-art review. J. Clean. Prod..

[20] Guo M., Zhang R., Du X., Liu P. (2024). A State-of-the-Art Review on the Functionality of Ultra-Thin Overlays Towards a Future Low Carbon Road Maintenance. Engineering.

[21] Roy S., Hossain Z. (2023). Effects of Aging on Multiscale Mechanistic Properties of Asphalt Binders. Int. J. Pavement Res. Technol..

[22] Xu B., Ding R., Yang Z., Sun Y., Zhang J., Lu K., Cao D., Gao A. (2023). Investigation on performance of mineral-oil-based rejuvenating agent for aged high viscosity modified asphalt of porous asphalt pavement. J. Clean. Prod..

[23] Yan Y., Hernando D., Roque R. (2019). A solvent free method to characterize the effect of recycled asphalt shingles on virgin asphalt binder. J. Clean. Prod..

[24] Behnood A. (2019). Application of rejuvenators to improve the rheological and mechanical properties of asphalt binders and mixtures: A review. J. Clean. Prod..

[25] Ran Y. (2018). Study on Performance and Mechanism of Multiplerecycled SBS Modified Asphalt. Master’s Thesis.

[26] Ding Y. (2017). Research on Aging and Recycling Performance of SBS Modified Asphalt. Master’s Thesis.

[27] Yi J., Wang Y., Pei Z., Xu M., Feng D. (2023). Mechanisms and research progress on biological rejuvenators for regenerating aged asphalt: Review and discussion. J. Clean. Prod..

[28] Enfrin M., Gowda A., Giustozzi F. (2024). Low-cost chemical modification of refined used cooking oil to produce long-lasting bio-asphalt pavements. Resour. Conserv. Recycl..

[29] Cao X., Quan Y., Deng M., Tang B., Kong L. (2024). Progress and Perspective of Bio-asphalt Preparation, Structural Characterization, and Rheological Properties. Energy Fuel.

[30] Chen Y., Chen Z., Xiang Q., Qin W., Yi J. (2021). Research on the influence of RAP and aged asphalt on the performance of plant-mixed hot recycled asphalt mixture and blended asphalt. Case Stud. Constr. Mater..

[31] Al-Qadi I.L. (2007). Reclaimed asphalt pavement—A literature review. Crack. Asph. Concr. Pavements.

[32] Baghaee Moghaddam T., Baaj H. (2016). The use of rejuvenating agents in production of recycled hot mix asphalt: A systematic review. Constr. Build. Mater..

[33] Zaumanis M., Mallick R.B., Frank R. (2013). Evaluation of Rejuvenator’s Effectiveness with Conventional Mix Testing for 100% Reclaimed Asphalt Pavement Mixtures. Transp. Res. Rec. J. Transp. Res. Board.

[34] Raouf M.A., Williams R.C. (2010). Temperature and Shear Susceptibility of a Nonpetroleum Binder as a Pavement Material. Transp. Res. Rec. J. Transp. Res. Board.

[35] Yang X., Mills-Beale J., You Z. (2017). Chemical characterization and oxidative aging of bio-asphalt and its compatibility with petroleum asphalt. J. Clean. Prod..

[36] Zeng M., Pan H., Zhao Y., Tian W. (2016). Evaluation of asphalt binder containing castor oil-based bioasphalt using conventional tests. Constr. Build. Mater..

[37] (2011). Standard Test Methods of Bitumen and Bituminous Mixtures for Highway Engineering.

[38] (2004). Technical Specification for Construction of Highway Asphalt Pavements.

[39] Rowe G.M., Sharrock M.J. (2011). Alternate Shift Factor Relationship for Describing Temperature Dependency of Viscoelastic Behavior of Asphalt Materials. Transp. Res. Rec. J. Transp. Res. Board.

[40] Yusoff N.I.M., Jakarni F.M., Nguyen V.H., Hainin M.R., Airey G.D. (2013). Modelling the rheological properties of bituminous binders using mathematical equations. Constr. Build. Mater..

[41] Ruan Y., Davison R.R., Glover C.J. (2003). An Investigation of Asphalt Durability: Relationships Between Ductility and Rheological Properties for Unmodified Asphalts. Pet. Sci. Technol..

[42] Sui C., Farrar M.J., Harnsberger P.M., Tuminello W.H., Turner T.F. (2011). New Low-Temperature Performance-Grading Method. Transp. Res. Rec..

[43] Sui C., Farrar M.J., Tuminello W.H., Turner T.F. (2010). New Technique for Measuring Low-Temperature Properties of Asphalt Binders with Small Amounts of Material. Transp. Res. Rec..

[44] Guo M., Yao X., Du X. (2023). Low temperature cracking behavior of asphalt binders and mixtures: A review. J. Road Eng..

[45] Guo M., Liang M., Liu H., Du X. (2023). Optimization and validation of waste bio-oil based high-performance rejuvenator for rejuvenating aged bitumen. Mater. Struct..

[46] Li C., Ma F., Fu Z., Dai J., Wen Y., Shi K. (2022). Using Cereclor plasticizer to modify the virgin asphalt binder: A case of rheological properties improvement. Constr. Build. Mater..

[47] Cao W., Li X. (2022). The Rejuvenating Potential of Plasticizers on Oxidatively Aged Asphalts: Rheological and Molecular Dynamics Perspectives. Polymers.

[48] (2004). Technical Specifications for Highway Asphalt Pavement Recycling.

[49] Blankenship P.B., Anderson M.A., King G.N. A Laboratory and Field Investigation to Develop Test Procedures for Predicting Non-load Associated Cracking of Airfield HMA Pavements. Airfield Asphalt Pavement Technology Program 2010, 06-01. AMEC Project No. 09-119-00948. https://www.eng.auburn.edu/research/centers/ncat/files/aaptp/Report.Final.06-01.

